# Combining Multisensory Cueing and Velocity-Based Training to Enhance Shot Put Performance in an F12 Para-Athlete: A Case Report

**DOI:** 10.3390/sports14050181

**Published:** 2026-05-01

**Authors:** Lawrence W. Judge, Exal Garcia-Carrillo

**Affiliations:** 1 Marieb College of Health and Human Services, Florida Gulf Coast University, Fort Myers, FL 33965, USA; 2 College of Health, School of Kinesiology, Ball State University, Muncie, IN 47306, USA; 3 Department of Physical Activity Sciences, Faculty of Education Sciences, Universidad Católica del Maule, Talca 3480112, Chile; exal.garcia@gmail.com; 4 Department of Physical Activity Sciences, Universidad de Los Lagos, Osorno 5290000, Chile

**Keywords:** athletes, athletic performance, resistance training, human physical conditioning, track and field, sports, sports for persons with disabilities

## Abstract

This case report documents the multi-season development of a 38-year-old elite F12 shot putter with macular degeneration (<10% functional vision) who improved from 13.00 m to a personal best of 14.41 m between 2021 and 2023. Athletes classified as F11–F13 compete with significant visual impairment that limits spatial feedback during rotational tasks, yet longitudinal evidence describing integrated training frameworks remains scarce. A 12-month macrocycle integrated phase-dependent velocity-based resistance training using mean concentric velocity targets (0.70–1.00 m·s^−1^) monitored via linear position transducers with a 10% velocity loss threshold, combined with structured auditory and tactile cueing, including metronome pacing and environmental anchors. High-volume warm-ups and prehabilitation addressed a prior L4–L5 disk herniation. The athlete achieved 14.41 m at the 2023 U.S. Para Athletics Trials, with TrackMan^®^-verified release velocity of 11.3 m·s^−1^. Bench throw velocity improved by 35.4% (0.65 to 0.88 m·s^−1^) and squat jump velocity improved by 22.9% (1.18 to 1.45 m·s^−1^), while post-session RPE remained manageable, indicating improved neuromuscular readiness and training tolerance. No lumbar symptom recurrence occurred. This case illustrates that integrating velocity autoregulation, multisensory stabilization, and injury-informed preparation can support meaningful performance gains in visually impaired throwers and offers an applied framework for coaches working with F11–F13 athletes.

## 1. Introduction

The primary determinants of shot put distance are release velocity, projection angle, and release height, all of which are fundamentally dependent on an athlete’s maximal strength and explosive power [1,2]. For elite throwers, implementing classical periodization with an emphasis on high-load strength training (≥80% one-repetition maximum [1RM]) is critical for maximizing speed–strength [3]. However, while this approach establishes the essential strength base, it traditionally relies on predetermined percentages of 1RM, which may not account for daily fluctuations in an athlete’s readiness and performance capability [4]. Velocity-based training (VBT) refines this paradigm by prescribing loads from instantaneous bar speed data, optimizing power outputs and fatigue management [5,6].

As a refinement of traditional load prescription models, VBT uses real-time bar speed feedback to regulate training intensity, volume, and fatigue [4,5]. Rather than relying solely on percentage-based loading, VBT enables practitioners to target specific neuromuscular qualities, such as maximal strength or explosive power, while accommodating day-to-day fluctuations in readiness [7]. Higher concentric velocities during ballistic resistance exercises have been closely linked to improved power output and competitive performance [8], supporting the use of velocity metrics as both a monitoring and autoregulatory tool. Recent applied work in Paralympic throwing has further demonstrated that high-volume potentiating warm-up strategies can enhance neuromuscular readiness and support injury risk management, underscoring the importance of integrated preparation models for para throwers [9].

For Para-athletes classified as F12, defined as having visual acuity ranging from LogMAR 1.50 to 2.60 and/or a visual field constricted to a diameter of less than 10 degrees [10], restricted visual feedback alters their interaction with the sporting environment. This challenge is particularly pronounced when adopting the rotational technique, characterized by high angular velocity and complex spatial orientation, as opposed to the linear glide technique [11]. While the glide provides a more stable postural reference, the rotational method requires maintaining dynamic balance during rapid centrifugal acceleration without fixed visual anchors, a deficit inherent in F12 athletes that often leads to spatial disorientation, since vision plays a critical role in postural control and proprioception [12], and visual impairment is considered a non-modifiable intrinsic risk factor for biomechanical alterations [13]. Consequently, this deficit complicates critical tasks such as spatial orientation, perception of field boundaries, and kinesthetic error correction during complex motor sequences [14,15]. Laboratory studies show altered kinematics and greater movement variability in blind throwers, who must rely on auditory and tactile cues for motor control and spatial updating [16], emphasizing the need for structured multisensory interventions to facilitate neuromuscular mapping. In the present case, the athlete’s visual impairment was attributable to early onset macular degeneration, characterized primarily by the degradation of central visual acuity with partial preservation of peripheral vision. This pattern is particularly consequential for rotational throwing tasks, as central vision is critical for fine spatial calibration, target alignment, and anticipatory motor correction. The relative loss of high-resolution focal input increases reliance on proprioceptive and auditory cues during high-velocity movement execution, thereby necessitating structured multisensory compensatory strategies including rhythm-based auditory cueing, consistent verbal cadence, tactile reference points, and stable environmental orientation anchors, to support movement timing, spatial awareness, and technical consistency under reduced visual input [17].

Although research in Para Athletics is expanding [18], longitudinal evidence describing integrated strength–power periodization specifically tailored for athletes with visual impairments (F11–F13) remains limited relative to the literature available for non-disabled throwing populations [18]. This persistent evidence gap hinders the development of evidence-informed training models for F12 athletes (e.g., VBT multisensory periodization with velocity targets/loss thresholds) and limits the generalizability of programming principles from non-disabled populations (e.g., percentage loading, visual instruction, and axial squatting [4,11,15]) to the unique sensory and biomechanical constraints faced by F12 athletes, including unstudied variables like metronome tempo, velocity loss injury, and VBT-to-release transfer [19,20,21]. To address this limitation, the purpose of this case report is to document the systematic implementation of an integrated training framework that combines velocity-based autoregulation VBT, multisensory motor stabilization strategies, and injury-informed load management over a 12-month macrocycle in an elite F12 shot putter. By detailing both performance outcomes and training process variables, this report aims to provide a translational, evidence-based framework for coaches and sport scientists working with visually impaired throwers, thereby extending current knowledge on constraint-adapted strength–power development in Para Athletics.

## 2. Materials and Methods

### 2.1. Participant Information

#### 2.1.1. Demographic and Anthropometric Data

The participant was a 38-year-old male elite Para Athletics shot putter (height: 183 cm, weight: 127 kg) competing in the F12 classification for visually impaired athletes. Body composition was estimated using a standardized 7-site skinfold protocol [22], yielding an estimated body fat percentage of approximately 20% and a calculated lean body mass of ~102 kg. The athlete was right-hand dominant and employed a rotational shot put technique adapted to accommodate visual impairment-related sensory constraints.

#### 2.1.2. Injury History

The athlete had a documented history of a single diagnosed injury: L4–L5 lumbar disk herniation sustained in 2018, which was fully resolved prior to the study period through targeted rehabilitation and progressive core stability training. Given this history, lower body resistance training strategies were intentionally selected to minimize axial spinal loading while preserving high-force and high-velocity stimulus. Specifically, belt squat-based loading was emphasized as the primary lower body strength modality, while traditional back squat exposure was limited and applied selectively (approximately two sessions per month) for neuromuscular monitoring and technical reinforcement rather than as a primary loading strategy. This load management approach was implemented to support performance development while maintaining spinal load tolerance across high-velocity training phases.

### 2.2. Ethical Considerations

The athlete provided written informed consent for participation and for the publication of performance and training data in accordance with ethical standards for case study research involving human participants.

### 2.3. Training Program

#### 2.3.1. Periodization and Load Management

Training was structured within a 12-month macrocycle progressing through General Preparation, Specific Preparation, and Peaking phases, aligned with the athlete’s international competition calendar and major championship targets. Prior to program construction, the training framework was informed by consultation with recognized experts in maximal strength and speed–strength development, ensuring alignment with contemporary evidence-based practices in elite throwing and power sport preparation. The General Preparation phase emphasized the restoration of movement capacity, foundational strength development, and technical re-patterning following injury and a transition from glide to rotational technique. The Specific Preparation phase prioritized strength–speed and speed–strength qualities directly supporting shot put performance, while the Peaking phase emphasized maximal power expression, neural freshness, and competition readiness.

Across ballistic resistance exercises, velocity targets were phase-dependent rather than static throughout the macrocycle. These targets were selected based on established load–velocity relationships, wherein lower velocities (≤0.75 m·s^−1^) correspond to higher relative loads (≥75% 1RM) and greater force production, while higher velocities (≥0.90 m·s^−1^) optimize power output and explosive intent [23]. During the General Preparation phase, mean concentric velocity targets were maintained within 0.70–0.85 m·s^−1^ to emphasize foundational force development. During the Specific Preparation phase, targets shifted to 0.80–0.95 m·s^−1^, reflecting increased emphasis on strength–speed qualities. In the Peaking phase, velocity targets were constrained to 0.85–1.00 m·s^−1^ to prioritize rapid force expression while minimizing residual fatigue near competition. Load prescription and progression were governed by these velocity thresholds rather than fixed percentage-based intensities [24]. This approach allowed daily adjustment of external load in response to neuromuscular readiness, mitigating excessive velocity loss while preserving training intent across the macrocycle. The 10% velocity loss threshold was selected based on evidence that lower thresholds (10–20%) produce comparable strength and power gains with significantly less fatigue accumulation compared to higher thresholds (≥30%), which is particularly important for athletes with a history of lumbar pathology [25,26]. Sets were discontinued when repetition velocity declined by more than 10% from the fastest repetition within the set. A conservative 10% velocity loss threshold was selected to preserve explosive force expression and limit neuromuscular fatigue accumulation, as greater velocity loss has been associated with increased mechanical degradation and central fatigue [27].

#### 2.3.2. Velocity-Based Training and Autoregulation

Bar velocity was monitored using linear position transducers (GymAware; see Section 2.4.1 for device specifications). Mean concentric velocity was recorded for each repetition and used as the primary autoregulatory variable guiding set termination and load adjustment. Sets were discontinued when repetition velocity declined by more than 10% from the fastest repetition within the set. A conservative 10% velocity loss threshold was selected to preserve explosive force expression and limit neuromuscular fatigue accumulation. While higher thresholds (e.g., 20%) are commonly used in hypertrophy-oriented protocols, greater velocity loss has been associated with increased mechanical degradation and central fatigue [28]. Given the athlete’s history of lumbar disc pathology and the elevated cognitive demand imposed by visually constrained rotational execution, minimizing fatigue-induced variability was prioritized to maintain movement integrity and spatial consistency across sessions [27].

This velocity loss criterion enabled consistent targeting of explosive force production while preventing excessive volume accumulation, particularly important given the athlete’s history of lumbar disk pathology. Load–velocity profiles were updated at the start of each training phase (every 4–6 weeks) using a standardized 4-load protocol (20%, 40%, 60%, and 80% of estimated 1RM) with three repetitions at lighter loads and one repetition at heavier loads, as previously recommended [23,29], to ensure that velocity targets remained aligned with the athlete’s evolving strength–power characteristics. The overall VBT framework was informed by Judge’s speed–strength model [30], emphasizing rapid force expression and movement intent as central drivers of performance adaptation.

#### 2.3.3. Technical Training and Multisensory Cueing

Technical training was conducted weekly and integrated with the physical preparation program to reinforce transfer of strength and power adaptations to sport-specific movement patterns. Video-based biomechanical analysis was used to evaluate rotational sequencing, ground contact timing, and trunk–hip separation, with particular attention to achieving a target release angle of 36–38°, consistent with biomechanical models of optimal shot put projection [31,32].

Given the athlete’s F12 visual impairment, technical instruction incorporated structured multisensory cueing to compensate for limited visual feedback. Tactile floor markers consisted of high-contrast chalk lines applied at fixed foot placement zones (entry, mid-rotation reference, and power position alignment) and circle boundary reference points. Chalk was selected due to its tactile detectability underfoot without altering surface compliance or friction characteristics. Unlike tape or raised materials, chalk did not introduce shear variability or modify proprioceptive feedback, thereby preserving ecological validity between training and sanctioned competition environments. Lines were refreshed between throws to maintain consistency of spatial reference A metronome set at 120 b·min^−1^ was selected to approximate the natural temporal cadence of the athlete’s rotational sequence during successful trials [33], as determined through frame-by-frame video analysis of pre-intervention throws. Specifically, the transition from entry to power position occurred over approximately 1.8–2.0 s, corresponding to a cadence near 2 Hz (120 beats per minute). This tempo provided an external auditory scaffold that reinforced consistent phase transitions without artificially accelerating movement tempo. Importantly, the selected frequency aligns with evidence suggesting that rhythmic auditory cueing near intrinsic movement frequency enhances motor timing stability and intersegmental coordination while minimizing cognitive load [19,20]. The 120 bpm tempo therefore functioned not as an imposed pacing constraint, but as a stabilizing temporal anchor synchronized with the athlete’s individualized movement rhythm. In addition, a metronome set at 120 b·min^−1^ provided an auditory rhythm cue to reinforce temporal sequencing from controlled entry to explosive delivery, supporting motor pattern stability and timing consistency [19,20]. These non-visual cues were systematically integrated into both technical and strength sessions to enhance sensorimotor integration and movement reproducibility.

Respecting the competition regulations of the International Paralympic Committee, all of the following adaptations were legally implemented. The athlete utilized a rotational shot put technique that was systematically adapted to account for visual impairment-related sensory limitations. Technical instruction and practice incorporated structured auditory and tactile spatial orientation strategies, including consistent verbal cadence, rhythm-based auditory cues, and fixed tactile reference points within the throwing circle. These strategies were designed to reinforce movement timing, spatial awareness, and phase transitions during throwing execution.

Beyond sensory cueing, specific biomechanical adaptations were implemented to stabilize rotational execution under constrained visual input. The initial wind-up amplitude was reduced by approximately 10–15% relative to conventional rotational models to limit excessive angular momentum accumulation during entry. The athlete maintained a comparatively more upright trunk orientation during the single-support and mid-rotation phases to minimize anterior center-of-mass displacement and reduce vestibular perturbation. The radius of rotation during the mid-phase was modestly shortened to improve angular velocity control and reduce spatial drift. Starting stance width was standardized to enhance proprioceptive reproducibility across trials. In addition, distal high-contrast environmental landmarks (mountain silhouettes beyond the sector and a fixed porch structure aligned with the intended power position) were used as macro-spatial orientation anchors. Collectively, these adaptations prioritized movement reproducibility and balance integrity over maximal angular displacement.

A common technical error in rotational shot put, particularly in athletes transitioning from glide to rotational technique, is excessive acceleration during the entry phase, often described colloquially as “going too fast out of the back of the ring.” In visually impaired throwers, this tendency may be amplified due to reduced central visual anchoring and diminished optical flow, which normally assist in regulating angular velocity and spatial orientation. Without stable visual reference, early over-acceleration can increase vestibular perturbation, compromise center-of-mass control, and degrade force transfer at delivery [13,34].

To mitigate this risk, the athlete was instructed to prioritize controlled entry tempo and posture integrity over maximal initial angular velocity. The reduced wind-up amplitude and upright trunk orientation functioned as deliberate constraints to prevent premature acceleration, preserve rotational sequencing, and enhance consistency at the power position. The metronome cue further reinforced controlled temporal progression, reducing the likelihood of early-phase overspeed that could destabilize subsequent movement phases.

#### 2.3.4. High-Volume Potentiating Warm-Ups

Each training session was preceded by a standardized high-volume potentiating warm-up designed to elevate core temperature, enhance neuromuscular activation, and reduce asymmetrical loading demands [9]. This performance enhancement is attributed to post-activation performance enhancement (PAPE), a physiological mechanism involving increased motor unit recruitment and myosin light chain phosphorylation following high-velocity contractions [35]. Warm-up protocols included upper body ballistic exercises (e.g., 3 × 15 medicine ball chest passes, band pulls) and dynamic trunk and hip mobility drills. The intent was to facilitate post-activation performance enhancement while minimizing injury risk in high-load and high-velocity training contexts, consistent with previous work in Para throwing populations [36]. Warm-up content and volume were maintained across phases to provide consistent preparatory stimuli, while intensity and movement specificity were adjusted in proximity to competition.

#### 2.3.5. Rehabilitation and Prehabilitation Integration

Given the athlete’s history of L4–L5 disk herniation, rehabilitation and prehabilitation strategies were systematically integrated into the weekly training structure to support both performance development and spinal load tolerance. Lower body resistance training primarily emphasized belt squat-based loading to permit high-force and high-velocity stimulus while minimizing axial spinal compression during strength–power development. Core stabilization exercises derived from the McGill “Big 3” were implemented three times per week to enhance spinal stiffness, trunk endurance, and force transmission during rotational and ballistic tasks [37]. Traditional back squat exposure was intentionally limited (approximately two sessions per month) and employed selectively for neuromuscular monitoring and technical reinforcement rather than primary loading. Lumbar and hip mobility exercises were prescribed to preserve segmental motion and movement efficiency without compromising spinal integrity.

In addition, the athlete underwent monthly physiotherapy sessions that included instrument-assisted soft tissue mobilization and dry needling to address residual myofascial restrictions. However, potential alterations in muscle tone arising from aberrant reflexes secondary to prior lumbar pathology—such as restricted ankle dorsiflexion—were not systematically assessed [35]. The athlete reported no lumbar symptoms during the study period and had no scars or tattoos in the treated areas that could influence local tone. Nonetheless, the absence of reflexive tone assessment constitutes a limitation, as addressing such dysfunction might have yielded additional performance gains.

#### 2.3.6. Representative Weekly Microcycle

To enhance translational clarity, Table 1 presents a representative weekly microcycle from the Peaking phase (4–6 weeks prior to major competition). This phase emphasized maximal power expression, movement precision, and neuromuscular freshness while maintaining spinal load tolerance.

### 2.4. Testing Tools and Performance Assessment (De 2.5 Original + Herramientas)

#### 2.4.1. Linear Position Transducer

The GymAware linear position transducer (Kinetic Performance Technology, Canberra, Australia) was used to measure mean concentric velocity (m·s^−1^) during bench press throws and squat jumps. This device has been shown to have high test–retest reliability for measuring movement velocity (ICC = 0.84–0.95) and is considered to be a reliable tool for tracking performance and monitoring fatigue [38]. This device has also been validated for measuring barbell velocity in resistance training exercises, demonstrating high reliability and minimal bias when compared to gold standard motion capture systems, including during ballistic movements such as the bench press [39].

#### 2.4.2. TrackMan Radar System

Competitive performance benchmarks were obtained using TrackMan^®^ (TrackMan A/S, Vedbæk, Denmark) radar-based ball flight analysis, which has been used previously to quantify release parameters in sport science research [40,41]. At the athlete’s personal best performance (14.41 m), release velocity was measured at 11.3 m·s^−1^, with a release height of 2.05 m and a projection angle of 35°. These kinematic variables were used as biomechanical reference targets to inform subsequent training emphasis and longitudinal monitoring.

#### 2.4.3. Rate of Perceived Exertion

Sessional rate of perceived exertion (RPE) was collected following resistance training sessions using the Borg 0–10 scale. The RPE values reflected perceived exertion during VBT-monitored training sessions occurring within ±7 days of competition and were not intended to represent competition effort. RPE was used as a complementary internal load measure to contextualize velocity outputs and monitor cumulative training stress across the macrocycle.

### 2.5. Training Background

The athlete began formal shot put training in 2017 and progressed to international-level competition by 2023. At the onset of the observation period (July 2021), the athlete’s personal best was 13.00 m, with a bench press throw mean concentric velocity of 0.65 m·s^−1^ and a post-session resistance training RPE of 5 (Borg 0–10 scale). Across the subsequent two competitive seasons, the athlete demonstrated progressive performance advancement, culminating in a sixth-place finish at the 2023 World Para Athletics Championships, a first-place finish at the 2023 Desert Challenge Games (14.14 m), and a personal best of 14.41 m at the 2023 U.S. Para Athletics Trials on 18 May 2023.

### 2.6. Training Environment and Support Structure

The athlete trained within an integrated performance support model involving coaching, strength and conditioning, and rehabilitation professionals. Warm-up procedures consisted of an extended dynamic potentiating protocol designed to enhance neuromuscular readiness, promote symmetrical loading, and reduce injury risk prior to high-velocity and high-load training sessions.

## 3. Results

The results presented below summarize longitudinal changes in competitive performance, explosive neuromuscular output, and internal load monitoring observed across the training macrocycle. Table 2, presented in reverse chronological order, provides a structured overview of competition outcomes, ballistic resistance training velocities, and post-session internal load measures recorded at key competitive time points. Collectively, these variables capture concurrent changes in sport-specific performance, resistance training velocity characteristics, and perceived training load, allowing for integrated evaluation of performance adaptation and training tolerance over time.

Across the macrocycle, improvements in competitive throwing distance were accompanied by progressive increases in ballistic resistance training velocity, reflected by increases in both bench press throw velocity (0.65 → 0.88 m·s^−1^) and squat jump velocity (1.18 → 1.45 m·s^−1^). Concurrently, post-session resistance training RPE demonstrated a modest downward trend, despite maintenance of high-velocity training targets. This pattern indicates improved tolerance to training demands and effective velocity-based load autoregulation rather than a reduction in training intensity.

TrackMan^®^ four-instrumented competition data collected during the personal-best performance on 18 May 2023 demonstrated a release velocity of 11.3 m·s^−1^, a release height of 2.05 m, and a projection angle of 35°. These kinematic parameters align with established biomechanical determinants of shot put performance and served as objective reference benchmarks for subsequent training and competition phases.

Across the 12-month macrocycle, no recurrence of lumbar symptoms or lumbar spine-related limitations were observed, and no training limiting or competition limiting musculoskeletal injuries occurred. Two minor, non-time-loss musculoskeletal complaints were documented: a transient low-grade groin strain involving the sweep leg during the rotational phase of throwing and mild throwing-side elbow discomfort associated with increased competition volume. Both conditions were managed conservatively through temporary load modification, targeted rehabilitation exercises, and close monitoring, and neither resulted in missed training sessions or competition withdrawal. These findings indicate that the injury-informed training and load management strategies employed were effective in supporting training continuity while mitigating clinically significant injury risk.

## 4. Discussion

**Figure 1:**
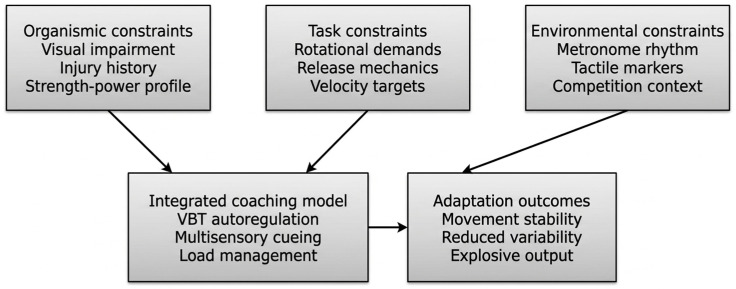
Conceptual framework for F12 shot put training integrating constraint-induced theory and cognitive load management. Synergy between visual constraint, multisensory cueing, VBT autoregulation, and injury-informed load management reduces cognitive load and movement variability while preserving explosive output.

Despite a growing body of literature on Para Athletics [18], longitudinal and intervention-based evidence remains scarce for several impairment classes, particularly athletes with visual impairments (F11–F13) [31]. This persistent evidence gap limits the development of evidence-informed training models and constrains the generalizability of strength–power programming principles derived primarily from able-bodied populations. The present case contributes to this emerging literature by documenting the systematic integration of velocity-based training, multisensory technical cueing, and injury-informed load management across a multi-season training period in an elite F12 shot putter, extending recent applied work in Paralympic throwing that emphasizes holistic preparation strategies [9]. Importantly, these adaptations were not implemented in isolation but reflected an integrated constraint-informed model wherein technical modification, velocity autoregulation, and multisensory stabilization strategies functioned synergistically to reduce movement variability while preserving explosive output under perceptual constraint. This approach aligns with the principles of constraint-induced movement therapy (CIMT), which posits that restricting compensatory options (here, unreliable visual feedback) forces the reorganization of motor execution through remaining sensory channels [42]. By systematically reducing visual input and introducing auditory/tactile anchors, the athlete’s nervous system was compelled to upregulate alternative sensory-motor pathways. Figure 1 presents a conceptual framework integrating these theoretical principles with the practical intervention components.

Following a season characterized by injury disruption and a continued transition from the glide to the rotational technique, the athlete demonstrated a meaningful expansion in competitive performance, improving from a seasonal best of 13.00 m to a personal best of 14.41 m. This improvement coincided with marked enhancements in explosive neuromuscular output, as reflected by increases in bench press throw velocity (0.65 → 0.88 m·s^−1^). These findings are consistent with prior research linking concentric velocity during ballistic resistance exercises to shot put performance and power expression [5,24], and they further support the utility of velocity metrics as both performance targets and autoregulatory monitoring tools in throwing populations. Importantly, these neuromuscular gains occurred within a training framework that prioritized fatigue management and movement quality, consistent with applied recommendations for Para throwers [9,43].

Critically, the observed performance gains cannot be attributed solely to improvements in strength–power capacity. This is consistent with evidence that rhythmic auditory cues reduce movement variability in visually impaired athletes by providing a temporal anchor for motor sequencing [19,20]. Future single-case experimental designs should directly compare throwing performance with versus without structured cueing to isolate this effect. Prior biomechanical investigations have demonstrated that visually impaired athletes exhibit greater movement variability and altered kinematic patterns during complex motor tasks, reflecting increased perceptual and coordinative demands [15,44]. By incorporating tactile reference points alongside rhythm-based auditory cues, the present intervention likely facilitated more consistent temporal sequencing of movement phases, reduced reliance on degraded visual input, and enhanced sensorimotor integration during both resistance training and sport-specific throwing tasks.

This interpretation is strongly supported by instructional research emphasizing rhythm as a central organizing principle in complex strength and athletic movements [45]. Research has demonstrated that rhythm-based instruction using auditory cues such as metronomes, music, and structured verbal cadence enhances technical consistency in power exercises by reinforcing the timing and coordination of movement phases rather than isolated positional cues [19]. Critically, this approach shifts instructional emphasis from visually dominated demonstrations to temporal patterning and movement flow, thereby supporting self-organized motor control. In the present case, rhythmic auditory pacing and consistent verbal cues likely functioned as compensatory control strategies, enabling the athlete to regulate phase transitions despite limited visual feedback. From a motor control perspective, rhythm acts as a higher-order constraint that anchors movement timing and reduces cognitive load by replacing high-frequency visual corrections with a stable temporal scaffold [31,46]. This shift from effortful to more automated motor execution is critical during rotational throwing, as it allows the athlete to focus on explosive force expression rather than internal monitoring of limb position [47]. The effectiveness of this auditory cueing is further supported by cognitive load theory, which demonstrates that imposing additional cognitive demands (e.g., visually tracking a target under impairment) degrades motor performance by taxing explicit learning systems [48]. By substituting a stable auditory scaffold for an unreliable visual one, the intervention reduced domain-specific cognitive load, freeing attentional resources for explosive force production [49].

The inclusion of high-volume potentiating warm-ups further supports a systems-based approach to performance enhancement [9]. Recent applied work in Paralympic throwing has demonstrated that structured, elevated-volume warm-up protocols can enhance acute force production while simultaneously supporting injury risk management in athletes with diverse impairment profiles [9]. In the present case, the consistent application of potentiating warm-ups may have contributed to improved neuromuscular readiness and symmetrical loading, particularly within the context of high-velocity resistance training and a history of lumbar disk pathology [50,51]. Notably, no lumbar symptom recurrence was observed across the macrocycle, underscoring the importance of embedding prehabilitation strategies within performance-oriented training models rather than treating injury prevention as a separate or secondary objective.

Collectively, these findings suggest that effective performance development in visually impaired throwers requires the deliberate integration of neuromuscular monitoring, rhythm-based multisensory technical instruction, and injury-informed load management, rather than reliance on isolated training components. While causal inference is inherently limited by the single-athlete design, the convergence of competitive performance outcomes, biomechanical data, and longitudinal monitoring strengthens the internal coherence of the observed adaptations and provides a compelling translational framework for Para Athletics practice.

### 4.1. Limitations

This case report is limited by its single-athlete design, absence of force plate-derived kinetic data, and the potential influence of age-related strength adaptations. We did not evaluate potential tone alterations from aberrant reflexes secondary to prior lumbar pain, which may influence distal function. Additionally, while velocity-based metrics provide meaningful insight into neuromuscular performance, they cannot fully characterize underlying force–time characteristics. Future research should prioritize controlled longitudinal designs involving multiple F11–F13 athletes and incorporate kinetic and neuromuscular assessments to validate and extend these findings.

### 4.2. Practical Applications

The findings of this case study provide Para track and field coaches with an applied framework for designing and delivering strength–power programs for visually impaired throwers that extend beyond visually dominated instructional models. Specifically, the results demonstrate that performance development in F11–F13 athletes can be effectively supported by integrating multisensory coaching strategies with objective neuromuscular monitoring, allowing coaches to maintain technical consistency and training intent despite constrained visual feedback.

From a coaching perspective, the use of rhythm-based auditory cues, structured verbal cadence, and tactile reference points offers a practical method for organizing complex movement sequences and stabilizing technique in high-velocity tasks. By anchoring movement execution to temporal and spatial cues rather than visual demonstration alone, coaches can facilitate more reliable motor patterning, reduce movement variability, and improve skill transfer from the weight room to the throwing circle. This approach is particularly valuable during periods of technical change, return-to-play following injury, or transitions between throwing styles. In addition, the integration of velocity-based training provides Para coaches with an objective tool for prescribing and autoregulating training loads in real time, independent of visual observation of bar speed or technique. Velocity feedback enables coaches to individualize loading, manage fatigue, and protect athletes from excessive training stress while still targeting sport-relevant explosive qualities. When combined with sessional RPE and injury-informed warm-up and prehabilitation strategies, this model supports safer progression and sustained performance development across a competitive season. For coaches seeking immediate implementation, Table 3 provides a tiered progression framework and classification-specific adjustments.

## 5. Conclusions

This case report demonstrates that an integrated framework combining VBT, multisensory cueing, and injury-informed load management supported meaningful performance gains in an elite F12 shot putter. Competitive distance improved from 13.00 m to 14.41 m, concurrent with increases in bench throw velocity (+35.4%) and squat jump velocity (+22.9%), without lumbar symptom recurrence. These findings illustrate that strength–power development, motor learning, and injury risk mitigation can be addressed simultaneously in visually impaired throwers. Although limited by its single-athlete design, this report provides a translational template for coaches working with F11–F13 athletes. Future studies should employ controlled designs with larger samples to validate these findings.

## Figures and Tables

**Table 1:** Representative Peaking phase microcycle (4 weeks pre-competition).

Day	Session Focus	Exercise Order	Exercise	Sets × Reps	Velocity Target/Intensity	Notes
Mon	Power emphasis	1	Dynamic warm-up (High-volume potentiation)	15–20 min	—	MB chest pass 3 × 15, band pulls
		2	Bench press throw	4 × 3	0.85–1.00 m·s^−1^	10% VL threshold
		3	Squat jump (concentric only)	4 × 3	0.90–1.00 m·s^−1^	LPT monitored
		4	Belt squat	3 × 4	~75–80% est. 1RM	Controlled eccentric
		5	McGill big 3 circuit	3 rounds	—	Curl-up, side plank, bird dog
Wed	Technical + speed	1	Warm-up	—	—	Rhythm integration
		2	Rotational throws	6 × 2	Comp weight	Metronome @ 120 bpm
		3	Overweight throws	3 × 2	+1 kg	Emphasis on sequencing
		4	Hip mobility + stability	—	—	Prehab emphasis
		2	Rotational throws	6 × 2	Comp weight	Metronome @ 120 bpm
Fri	Neural priming	1	Warm-up	—	—	Reduced volume
		2	Bench throw	3 × 2	0.90–1.00 m·s^−1^	Stop at fastest rep
		3	Belt squat (light)	3 × 3	~65–70%	Speed intent
		4	McGill big 3	2 rounds	—	Maintenance volume

**Table 2:** Longitudinal evolution of competitive performance, explosive power, and perceived exertion across the training macrocycle.

Timepoint	Competition (Place)	Result (m)	Δ% (dist)	SJV (m·s^−1^)	Δ% (SJV)	BTV (m·s^−1^)	Δ% (BTV)	RPE
July 2021	Baseline (Initial)	13.00	—	1.18 *	—	0.65	—	5.0
18 May 2023	U.S. Para Trials (1st)	14.41	+10.8%	1.45	+22.9%	0.88	+35.4%	6.0
26 May 2023	Desert Challenge (1st)	14.14	+8.8%	1.38	+16.9%	0.82	+26.1%	7.0
10 July 2023	World Champs (6th)	13.42	+3.2%	1.18	0.0%	0.65	0.0%	7.5
22 November 2023	Para Pan Am (2nd)	13.68	+5.2%	1.25	+5.9%	0.71	+9.2%	7.0

Note. Δ% indicates the percentage of change relative to the July 2021 baseline values; BTV = mean concentric bench throw velocity; SJV = mean concentric squat jump velocity; RPE = post-session rate of perceived exertion assessed using the Borg CR-10 scale. Values were recorded during VBT-monitored sessions occurring within ±7 days of sanctioned competitions. * Baseline SJV value was recorded during a baseline testing session and not in immediate proximity to a sanctioned competition.

**Table 3:** Practical adjustments for coaches working with F11–F13 shot putters.

Issue	Recommended Adjustment
Novice progression (F11–F13)	Seated throws → standing throws with tactile anchors → full rotational throws
Shoulder pain	Reduce overhead volume; substitute incline bench press throws (30–45°); increase band external rotation
Pre-competition anxiety	120 bpm metronome during warm-up throws; breathing cues synchronized with entry phase
F11 (no light perception)	Tactile circle boundaries (rope/raised chalk); verbal cadence for every phase
F13 (higher visual function)	High-contrast visual markers; can use more visually guided feedback; still need auditory anchoring

## Data Availability

The data presented in this study are not publicly available. The dataset consists of individualized athlete training records, coaching logs, and performance monitoring data generated within a high-performance training environment. Due to the sensitive and proprietary nature of these training data, as well as considerations related to athlete privacy and performance confidentiality, the data are not available for external access or sharing. However, aggregated or anonymized data may be made available from the corresponding author upon reasonable request and with permission from the athlete.

## Abbreviations

The following abbreviations are used in this manuscript:

1RM	one-repetition maximum
BTV	bench throw velocity
F12	Classification for Athletes with Visual Impairment
LPT	linear position transducer
RPE	rate of perceived exertion
SJV	squat jump velocity
VBT	velocity-based training
VL	velocity loss
